# HDAC6 regulates human erythroid differentiation through modulation of JAK2 signalling

**DOI:** 10.1111/jcmm.17559

**Published:** 2022-12-28

**Authors:** Pascal Vong, Kahia Messaoudi, Nicolas Jankovsky, Cathy Gomilla, Yohann Demont, Alexis Caulier, Guillaume Jedraszak, Julien Demagny, Stefan Djordjevic, Thomas Boyer, Jean Pierre Marolleau, Jacques Rochette, Hakim Ouled‐Haddou, Loïc Garçon

**Affiliations:** ^1^ HEMATIM UR4666 Université Picardie Jules Verne Amiens France; ^2^ Service d'Hématologie Biologique Centre Hospitalier Universitaire Amiens France; ^3^ Service des Maladies du Sang Centre Hospitalier Universitaire Amiens France; ^4^ Laboratoire de Génétique Constitutionnelle Centre Hospitalier Universitaire Amiens France

**Keywords:** HDAC, human erythropoiesis, JAK2 signalling

## Abstract

Among histone deacetylases, HDAC6 is unusual in its cytoplasmic localization. Its inhibition leads to hyperacetylation of non‐histone proteins, inhibiting cell cycle, proliferation and apoptosis. Ricolinostat (ACY‐1215) is a selective inhibitor of the histone deacetylase HDAC6 with proven efficacy in the treatment of malignant diseases, but anaemia is one of the most frequent side effects. We investigated here the underlying mechanisms of this erythroid toxicity. We first confirmed that HDAC6 was strongly expressed at both RNA and protein levels in CD34^+^‐cells‐derived erythroid progenitors. ACY‐1215 exposure on CD34^+^‐cells driven in vitro towards the erythroid lineage led to a decreased cell count, an increased apoptotic rate and a delayed erythroid differentiation with accumulation of weakly hemoglobinized immature erythroblasts. This was accompanied by drastic changes in the transcriptomic profile of primary cells as shown by RNAseq. In erythroid cells, ACY‐1215 and shRNA‐mediated HDAC6 knockdown inhibited the EPO‐dependent JAK2 phosphorylation. Using acetylome, we identified 14‐3‐3ζ, known to interact directly with the JAK2 negative regulator LNK, as a potential HDAC6 target in erythroid cells. We confirmed that 14‐3‐3ζ was hyperacetylated after ACY‐1215 exposure, which decreased the 14‐3‐3ζ/LNK interaction while increased LNK ability to interact with JAK2. Thus, in addition to its previously described role in the enucleation of mouse fetal liver erythroblasts, we identified here a new mechanism of HDAC6‐dependent control of erythropoiesis through 14‐3‐3ζ acetylation level, LNK availability and finally JAK2 activation in response to EPO, which is crucial downstream of EPO‐R activation for human erythroid cell survival, proliferation and differentiation.

## 1 INTRODUCTION

Numerous studies have highlighted the role of post‐translational modifications in the regulation of cell proliferation, differentiation and death.^1^, ^2^ Among these modifications, acetylation modifies the physico‐chemical properties of proteins and modulates their activity, stability, localization and affinity for partner proteins. Acetylation and deacetylation are catalysed by Histone Acetyl‐Transferases (HAT) and Histone Deacetylases (HDACs), respectively. Through the deacetylation of a wide variety of functional and structural, nuclear and cytoplasmic proteins, HDACs modulate multiple cellular processes.^3^, ^4^, ^5^ In humans, there are 18 HDACs classified into four classes‐I, IIa, IIb, III and IV, according to their structural homology. The first class includes nuclear HDACs capable of deacetylating histones and transcription factors (HDAC1, 2, 3 and 8). Enzymes in the second class can shuttle between cytoplasmic‐nucleus compartments. Class II is subdivided into two subgroups: IIa, consisting of HDAC4, 5, 7 and 9 and IIb, consisting of HDAC6 and HDAC10. HDAC11 is the only member of HDAC class IV. Several studies have shown that HDAC expression and activity can be altered in several types of cancers where they stimulate cell proliferation and survival, promote angiogenesis and inhibit apoptosis. Currently, various HDAC inhibitors are being evaluated in clinical trials for the treatment of solid and haematological cancers as well as cardiac and neurodegenerative pathologies.^6^, ^7^, ^8^, ^9^, ^10^, ^11^ Ricolinostat (ACY‐1215) is a selective inhibitor of HDAC6 (HDAC6i) that has been shown to be of value in treating malignant diseases such as multiple myeloma, lymphoma and solid cancers in phase I and II clinical trials.^12^, ^13^, ^14^, ^15^ Among histone deacetylases, HDAC6 is unusual in its cytoplasm location which facilitates its role in deacetylating various non histone protein targets such as α‐tubulin, the chaperone protein HSP90 or Cortactin (CTTN)^16^, ^17^, ^18^ . After exposure to chemical inhibitors, hyperacetylation of HDAC6 targets blocks the cell cycle, inhibits proliferation and induces apoptotic death. Clinical trials highlighted frequent secondary effects observed with selective HDAC6i as monotherapy or in combination with other molecules, related to hyperacetylation of proteins in off target non‐malignant cells. Among these off‐target effects, one of the most frequent is haematological.^12^, ^13^, ^14^, ^15^, ^19^, ^20^ Thrombocytopenia, occurring in around 13% of patients, is related to the inhibition of the HDAC6‐CTTN axis, which plays a positive regulatory role in human megakaryocyte (MK) maturation. Indeed, CTTN hyperacetylation through HDAC6 inhibition decreases its ability to bind to F‐actin, which inhibits MKs terminal differentiation.^21^ Anaemia is another frequent HDAC6i‐induced toxicity, occurring in 9%–20% of patients.^12^, ^15^, ^19^ However, its mechanism is poorly understood. In mice, the HDAC6‐mDia2 axis is involved in the terminal stages of erythropoiesis, controlling cytokinesis and enucleation. Indeed, in mouse erythroblasts, mDia2 deacetylation through HDAC6 leads to its activation, contractile actin ring (CAR) formation and cytokinesis facilitating the extrusion of the nucleus, this axis being altered after HDAC6 knockdown or inhibition.^22^ Whether such mechanism also occurs during human erythropoiesis and is involved in the erythroid toxicity associated with HDAC6i is unknown.

We present here an extensive study of HDAC6 expression and function during in vitro erythroid differentiation of primary human CD34^+^ cells and show its crucial involvement in regulating the JAK2 signalling pathway, which is downstream EPO‐R activation for erythroid cell survival, proliferation and differentiation.

## 2 METHODS

### 2.1 In vitro culture of human CD34 ^+^ erythroid cells and UT7/EPO cell line

CD34^+^ progenitor cells were isolated by positive selection kit (MiltenyiBiotec) using immunomagnetic beads cell‐sorting system (AutoMACS; MiltenyiBiotec) from healthy leukapheresis samples (from allogenic stem cell transplantation donors), in agreement with our institutional ethics committee. CD34^+^ cells were cultivated as recently described^23^ from day (D)1 to D6 in Iscove's modified Dulbecco's medium (IMDM) (Biochrom) supplemented with glutamine (Eurobio), containing 2% human AB serum (H2B), 100 IU/ml penicillin (Eurobio), 100 μg/ml streptomycin (Eurobio), 500 μg/ml human holo‐transferrin (Sigma‐Aldrich), 10 μg/ml recombinant human insulin (Sigma‐Aldrich), 2 IU/ml heparin (Braun), 100 ng/ml human stem cell factor (SCF) (MiltenyiBiotec) and 3 IU/ml erythropoietin (EPO) (Roche). From D7 to D20, cells were culture in the same medium but after SCF removal. UT7/EPO cells were maintained in α‐minimum essential medium (MEM) (Dominique Dutscher) supplemented with 10% FCS (Eurobio) and 2 IU/ml EPO as recently described.^24^

### 2.2 Flow cytometry (FCM)

Cells were washed and stained with appropriate panels of conjugated antibodies in 1X PBS plus 2 mM EDTA and 0.5% BSA on ice. All conjugated antibodies and corresponding isotypes were supplied from MiltenyiBiotec. In all conditions, exclusion of non‐viable cells was assessed using 7‐amino‐actinomycin D (7‐AAD, MiltenyiBiotec) or DAPI (MiltenyiBiotec). Apoptosis was assessed using the Miltenyi Biotech kit according to the manufacturer's instructions. Phosphoflow experiments were performed on cells starved overnight without EPO, then re‐stimulated with 1 IU/ml EPO for 5 min, before fixation, permeabilization and staining (cf. Table S1 for antibody references). Acquisition was performed on a MACSQuant flow cytometer and analysis on FlowJo software.

### 2.3 Flow imaging

Flow imaging was performed on ImageStream^®X^ Mark II (Amnis/Luminex) using INPIRE™ software (200.1.388.0). 1.10^6^ cells in 50 μl 1X PBS were stained with human Anti‐HDAC6 antibody. Cell analyses were performed on at least 10,000 cells at 60× magnification. Analysis was performed using IDEAS™ software (6.2.64.0).

### 2.4 Lentiviral particles production and UT7/EPO cell transduction

ShRNA directed against HDAC6, described used in a previous report, were a kind gift of Dr Najet Debili (IGR, INSERM).^21^ Viral production and cell transduction were ensured as recently described.^24^ UT7/EPO cells were infected using a MOI of 10; cells were washed 2 times in 1X PBS 48 hours after transduction and GFP positives cells were sorted using a FACSAria II (Becton Dickinson).

### 2.5 Immunoblots

Cells were harvested at 10^6^/ml per condition, washed in 1X PBS and lysed on ice in RIPA buffer (Sigma) containing protease and phosphatase inhibitors cocktail (Thermofisher). For phosphoblot experiments, cells were starved overnight without EPO, before re‐stimulation with 10 IU/ml EPO for 5 min. For protein localization, nuclear and cytoplasmic extracts were purified according to standard procedure. Lysates were gently sonicated, centrifuged and protein concentration was quantified using Bradford assay (Interchim). After standard procedures of SDS‐polyacrylamide gel electrophoresis, nitrocellulose membrane transfer and 5% non‐fat milk blocking, blotting was performed using 1/1000 diluted primary antibodies (references in Table S2 **)**. Bound primary antibodies were detected using anti‐mouse (Sigma‐Aldrich) or anti‐rabbit (Thermofisher) horseradish peroxidase‐ conjugated secondary antibody. Revelation was performed by enhanced chemiluminescent substrate, SuperSignal West Femto or Pico Substrates (Thermofisher), and signals were visualized using Chemidoc Device (BioRad). Images were analysed using ImageLab software.

### 2.6 Protein extraction for immunoprecipitation experiments

Protein acetylation was studied using immunoprecipitation (IP) experiments. Cells were lysed on ice in an IP buffer (1%NP40, 150 mM NaCl, 5 mM EDTA, 65 mM Tris HCl pH 8, 50 mM HEPES, 3% glycerol, 1 mM orthovanadate, 10 mM sodium butyrate, 1% Phosphatase inhibitors Cocktail and 1% Protease Inhibitors Cocktail) for 20 min. Cell lysates were also sonicated three times and homogenized with a pellet mixer (VWR®). After centrifugation of cell lysates at 9000 *g* for 15 min at 4°C, the supernatants were collected and the protein concentrations were determined by colorimetric based assays (NanoPhotometer® Pearl).

### 2.7 Immunoprecipitation, acetylation and protein–protein interactions analysis by immunoblot

Protein acetylation and protein–protein interactions were studied using immunoprecipitation (IP) experiments. Cell lysates were incubated with an antibody directed against the protein of interest or an IgG control antibody (2 μg) overnight at 4°C. On the following day, immunocomplexes were incubated with 50 μl μMACS Protein G Microbeads (MiltenyiBiotec) during 45 min at 4°C. The magnetically tagged immunocomplexes were then isolated in μColumns (MiltenyiBiotec) according to the manufacturer's instructions. Subsequently, an immunoblotting was performed to detect and characterize the acetylation status of the protein of interest or to identify its partner proteins within the same complex.

### 2.8 RQ‐PCR

Total RNA from fresh cells was isolated, treated with DNAse I, using the RNeasy Mini Kit (Qiagen). RNA quantity was determined with the NanoDrop ND‐1000 Spectrophotometer (Thermo Fisher Scientific). For each sample, 500 ng of RNA was subjected for reverse transcription reaction (Reverse Transcription Kit, Thermofisher). For the expression ratios of target genes versus GAPDH housekeeping gene, real time PCR was performed by power SYBR green assay (Thermofisher). Primer sequences are listed Table S3. Experiments were realized in the Quant Studio 7 Detection Device (Applied Biosystems), including dissociation curves setting. The comparative C_T_ method was used for quantification of gene expression.

### 2.9 Cytospin for cytology, immunofluorescence microscopy

50.10^3^ cells were cytospun (Cytospin3, Shandon) onto glass slides (Matsunami). Cytological analysis was performed after May‐Grünwald Giemsa (MGG) staining (Sigma) according to manufacturer's instruction. For fluorescent microscopy, 50.10^3^ cells were fixed with 1% paraformaldehyde (PFA, Thermofisher), permeabilized (Triton X100 0,1%, Sigma), washed by centrifugation (300 *g*, 5 min) resuspended in 100 μl 1X PBS‐ 5%BSA. Cells were incubated overnight at 4°C with an anti‐HDAC6 mouse primary antibody (1/100, sc‐28,386), then washed by centrifugation (300 *g*, 5 min) and resuspended in 100 μl 1X PBS‐ 5%BSA. Staining was performed with an Alexa Fluor® 647 anti‐mouse antibody (Thermofisher, A‐21235) at room temperature for 1 h. After gentle washing followed by cytospin (300 g, 5 min), coverslip‐attached erythroblasts were mounted onto glass plates using ProLong Gold Antifade Mountant with DAPI (Thermofisher). Images were taken using an Axio Imager M2 ApoTome microscope running ZEN software (Zeiss).

### 2.10 Reagents

ACY‐1215 was purchased from Selleckchem. Dilutions were prepared in dimethylsulfoxide (DMSO).

### 2.11 RNA sequencing

Sequencing and bioinformatics analysis were performed by the Genomics and Bioinformatics facility (GBiM) from the U 1251/Marseille Medical Genetics lab. mRNA‐Seq was performed on total RNA samples from primary erythroid cells at D10 of differentiation, with or without exposure to ACY‐1215, in triplicate. Before sequencing, the quality of total RNA samples was assessed by using the Agilent bioanalyzer (Agilent): only RNAs with RNA Integrity Numbers (RIN) above 8 were deemed suitable for sequencing and used for library preparation. For each sample, a library of stranded mRNA was prepared from 500 ng of total RNA after capture of RNA species with poly‐A tail poly(A), using the Kapa mRNA HyperPrep kit (Roche), following the manufacturer's instructions. Indexed libraries were pooled and sequenced on an Illumina NextSeq 500 platform (75 bp paired‐end sequencing).

### 2.12 Data processing and differential gene expression analysis

The quality of sequencing reads was assessed using fastQC.^25^ Raw sequencing reads were mapped to the human reference genome (Homo Sapiens genome assembly GRCh38 [hg38]) using STAR v2.5.3a^26^ and bam files were indexed and sorted using Sambamba v0.6.6. After mapping, Number of reads per feature (GENCODE v34 annotations) was determined using Stringtie v1.3.1c.^27^ Differential gene expression analysis was performed using a Wald test thanks to the DESeq2 package.^28^ *p*‐values were adjusted for multiple testing using the method by Benjamini and Hochberg: Only transcripts with an adjusted *p*‐value (FDR, False Discovery Rate) below 0·05 were considered as significantly differentially expressed. Differentially Expressed Genes (DEG) were visualized in the form of heatmaps generated using the Pheatmap R package, or volcano plots, representing the Log_2_FC (log2 of the expression fold change) and the adjusted *p*‐value, prepared using the EnhancedVolcano R package. Preranked Gene set Enrichment Analysis (GSEA) of DEGs was achieved with the GSEA software (Version 4.2.0). Preranked GSEA was performed using Kolmogorov–Smirnov test and 1000 gene‐set permutation.

### 2.13 Statistical analysis

Statistical analyses were performed using two‐tailed *p* value and parametric tests. Statistical significance used was *α* = 0.05. For quantitative variables, we used Student's *t*‐test or one‐way anova test and Tukey post‐hoc analysis for multiparametric analysis. All numeric values as mean values ± SD.

## 3 RESULTS

### 3.1 HDAC6 is expressed and located in the cytoplasm of erythroid cells during in vitro human erythroid differentiation

**FIGURE 1:**
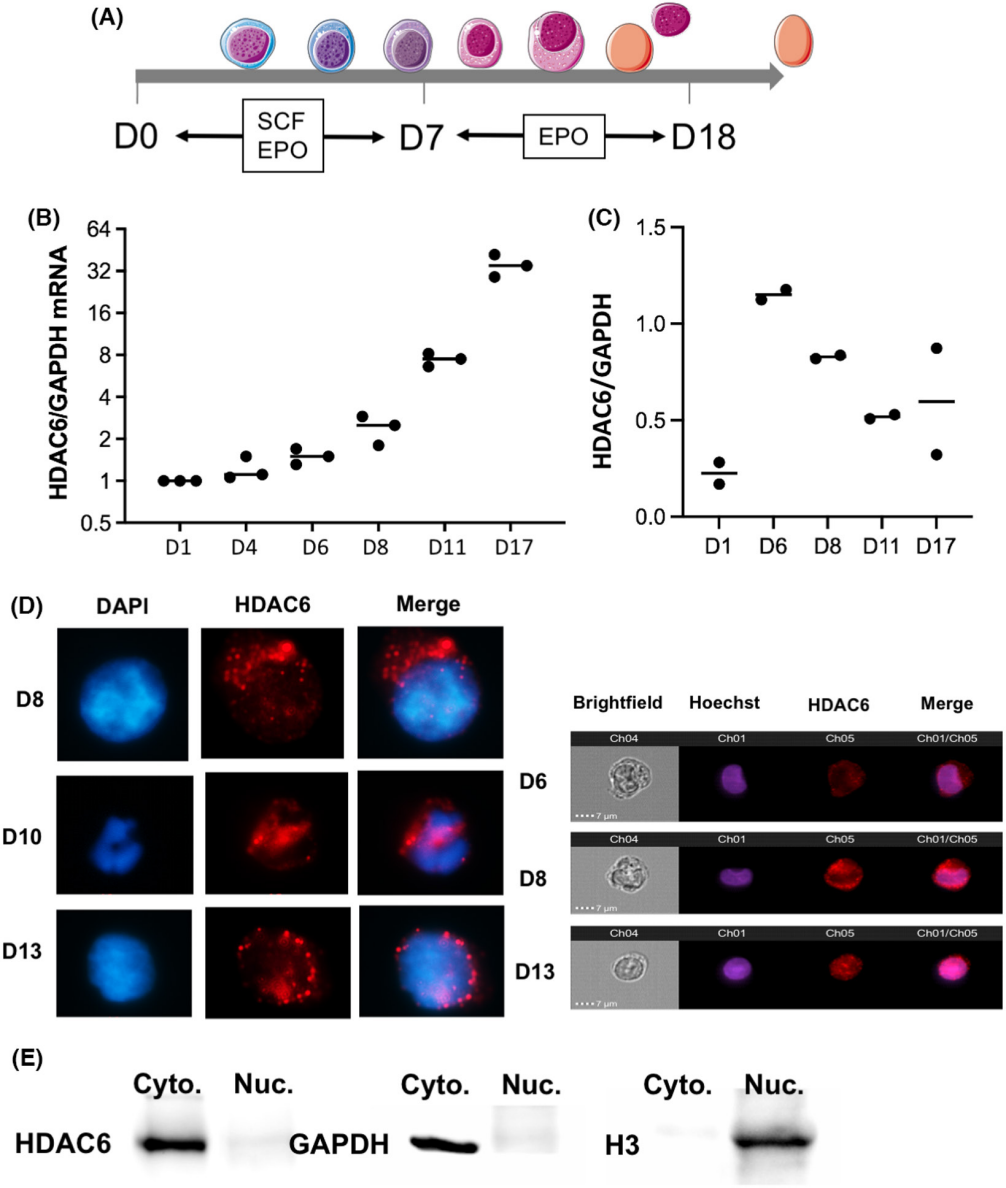
HDAC6 is expressed during human erythroid differentiation and is localized in the cytoplasm of erythroid cells. (A) Scheme representing the in vitro differentiation protocol used in this study and the phenotype of erythroid cells at D6–D8 and D10 corresponding respectively to ACY‐1215 exposure and phenotype analysis. (B) RQ‐PCR showing *HDAC6* expression at different time points of in vitro erythroid differentiation of CD34^+^ cells from apheresis (*n* = 3). GAPDH was used as a housekeeping gene. (C) HDAC6 expression at protein level quantified by Western Blot analysis using the same differentiation assay (*n* = 2). (D) HDAC6 localization in primary erythroid cells at D6, D8, D10 and D13 of in vitro differentiation using immunofluorescence (left, *n* = 3) and flow imaging (right, *n* = 1). (E) Western Blot on nuclear and cytoplasmic fractions on D8 primary cells undergoing erythroid differentiation confirming the exclusive HDAC6 cytoplasmic localization. Histone H3 and GAPDH were used as positive controls of nuclear and cytoplasmic fractions respectively (*n* = 2).

We used a previously published model of in vitro erythroid differentiation of CD34^+^ cells purified from apheresis^23^ (Figure 1A and Figure S1A). We first quantified HDAC6 expression sequentially during erythroid differentiation and observed a progressive increase in HDAC6 RNA expression raising from Day (D) 11 to D18 (Figure 1B and Figure S1B). At the protein level, we observed an earlier increase in HDAC6 expression at D6 of differentiation followed by a progressive decrease in the late stages of differentiation (Figure 1C and Figure S1C**)**. This expression pattern and apparent discrepancy between RNA and protein level are in agreement with proteomic data of human erythropoiesis published by Gautier et al.^29^ Using indirect immunofluorescence and flow imaging to assess HDAC6 cellular localization during erythroid differentiation, we observed that HDAC6 expression was mainly cytoplasmic at D8 arguing for non‐histone and cytoplasmic targets at this step of erythropoiesis (Figure 1D). We confirmed this data using Western Blot after separation of the nuclear and cytoplasmic fractions of proteins using Histone H3 and GAPDH as controls **(**Figure 1E).

### 3.2 Effects of HDAC6 inhibition on the human in vitro erythroid differentiation

**FIGURE 2:**
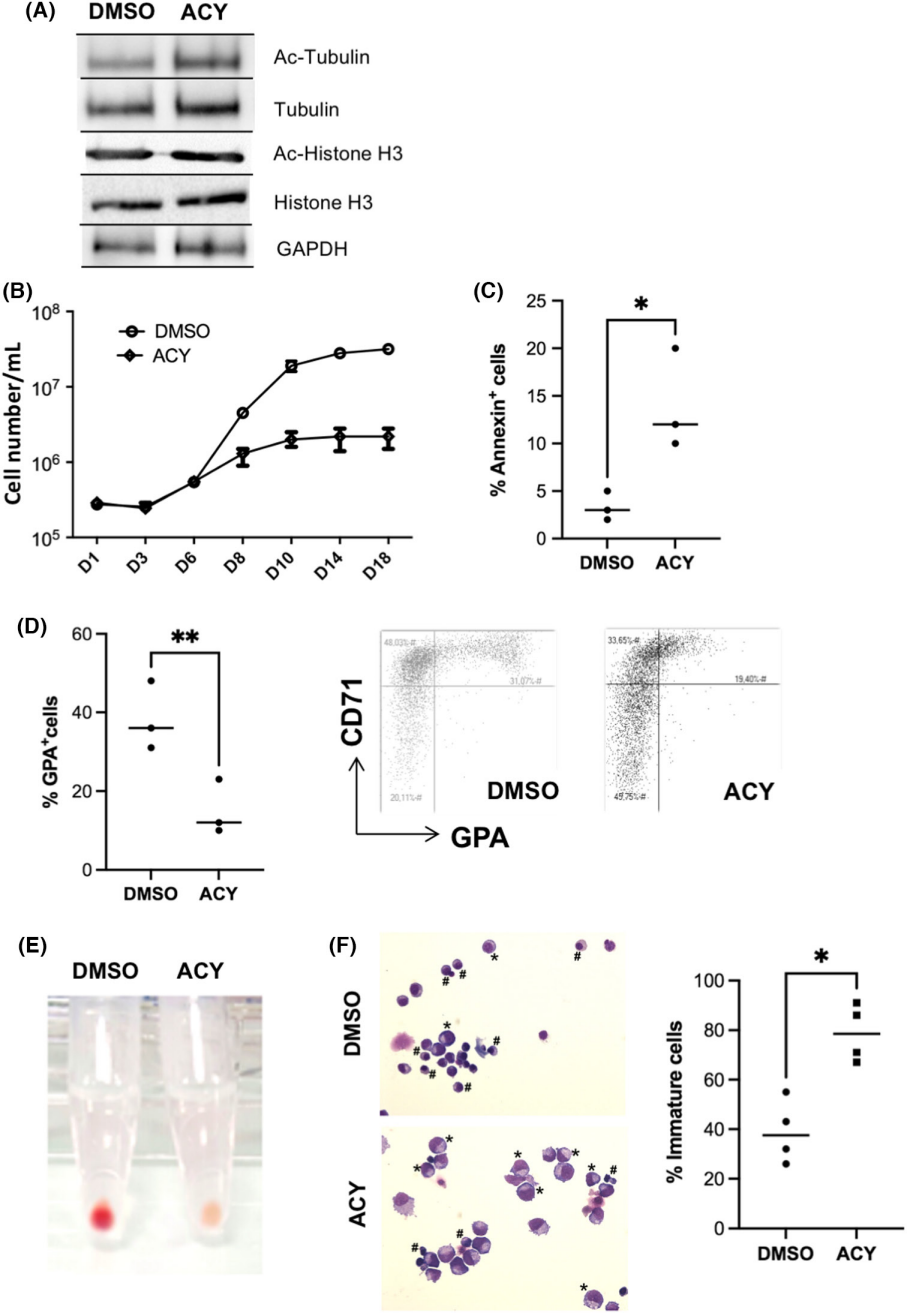
Consequences of HDAC6 chemical inhibition during in vitro human erythroid differentiation (A) A representative immunoblot showing the acetylation level of Histone H3 (used as negative control since not an HDAC6 target) and acetylation level of α‐tubulin (positive control, since a known cytoplasmic HDAC6 target) after HDAC6i exposure in human primary erythroid cells (*n* = 3). GAPDH is the same internal control for both. Total Histone H3 and Total α‐tubulin images were performed after stripping of each corresponding membrane part. All component parts of blot were indicated by dividing lines. (B) Comparative proliferation curves of human primary cells driven towards erythroid differentiation exposed to 0.5 μM ACY‐1215 or DMSO, *n* = 3. (C) Increased apoptosis in D10 erythroid progenitors exposed to 0.5 μM ACY‐1215 in comparison with DMSO: % Annexin‐V ^+^ cells, DMSO versus ACY: 3.33 (±1.5) versus 14 (±5.3), *n* = 3, *p* < 0.05. (D) Decreased percentage of GPA^High^ cells at D10 of erythroid differentiation assessed by FCM after exposure to 0.5 μM ACY‐1215 vs. DMSO. Right: CD71/GPA dot plot obtained at D10 in one representative experiment. % of GPA^High^ cells at D10, DMSO versus ACY: 38 (±8.7) versus 15 (±7), *n* = 3, *p* < 0.01. (E) Pictures of the red cell pellet obtained at D10 after DMSO and 0.5 μM ACY‐1215 exposure (one representative experiment, *n* = 4). (F) Cytospin after May Grünwald Giemsa staining showing the D10 erythroblasts morphology after DMSO and 0.5 μM exposure; immature cells are identified by *; left: image from one representative experiment; right: histograms showing the % of immature erythroblasts (ProE+ BasoE) in the two conditions: DMSO versus ACY‐1215: 39% (±12.8) versus 78.7% (±11.6), *n* = 4, *p* < 0.05.

We cultured CD34^+^‐cells into in vitro driven erythroid differentiation in the presence of the HDAC6 pharmacological inhibitor, ACY‐1215 from D6 to D10. We first evaluated the specificity of HDAC6i using Western blot by assessing the level of acetylation of tubulin (a primary target of HDAC6), and its selectivity by the level of acetylation of histone H3. We observed that 0.5 μM ACY‐1215 gave the best result in terms of HDAC6 inhibition, leading to an increased acetylated α‐tubulin without affecting histone H3 acetylation (Figure 2A). When erythroid‐differentiated CD34^+^ cells were exposed to ACY‐1215, HDAC6 pharmacological inhibition did not affect the clonogenic potential of erythroid progenitors (Figure S2A). When added at D6, ACY‐1215 significantly decreased cell count at D8 and D10 (Figure 2B) and increased the apoptotic rate as shown by Annexin‐V staining (Figure 2C). We evaluated the effects of HDAC6 inhibition on erythroid differentiation using flow cytometry (CD71/GPA staining) and cytology. We observed at D10 a delay in erythroid differentiation with a decreased percentage of GPA^High^ cells (Figure 2D). This delay in differentiation resulted in a decrease in haemoglobin synthesis with a cell pellet remaining barely red at D10 (Figure 2E). Accordingly, MGG staining at D10 revealed an increase in immature erythroblasts at the expense of mature cells (Figure 2F and Figure S2B). All together, these experiments showed that HDAC6i led to a delay in early erythroid precursors, downstream the CFU‐E stage.

### 3.3 Delay in erythroid maturation induced by ACY‐1215 occurs at transcriptomic level and is associated with a global modification of erythroid cell transcriptome

**FIGURE 3:**
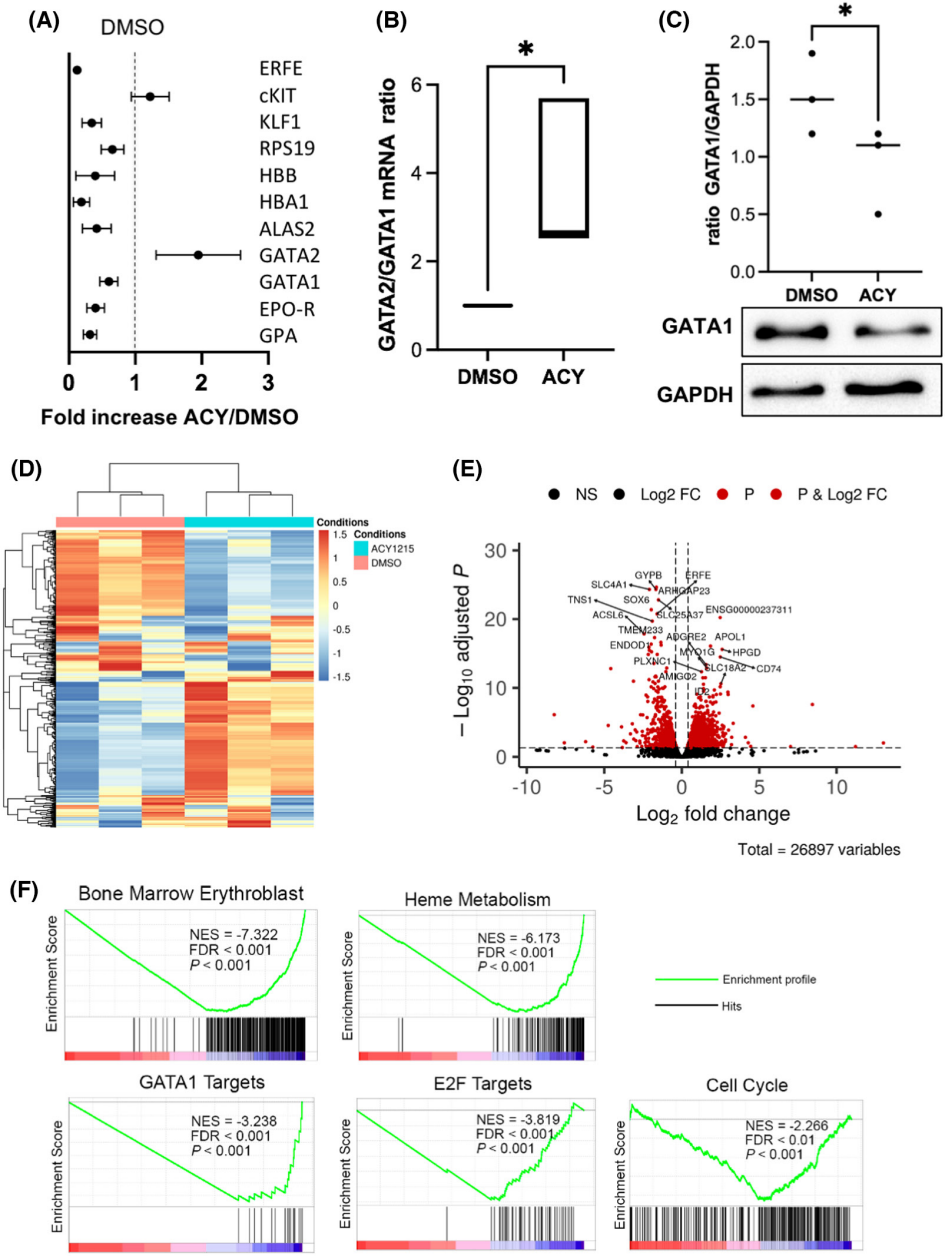
Global transcriptomic modulation induced by HDAC6 inhibition in primary cells. (A) RQ‐PCR showing the differential expression of 11 genes involved during erythroid differentiation between primary erythroid cells exposed to DMSO or ACY‐1215. The figure represents the fold increase in the ACY‐1215 conditions relative to DMSO, *n* = 4. (B) *GATA2/GATA1* ratio at RNA level assessed by RQ‐PCR in primary erythroid cells between the DMSO and the ACY‐1215 conditions, *n* = 4, *p* < 0.05. (C) GATA1 decrease at protein level assessed by Western blot in primary erythroid cells exposed to ACY‐1215 comparatively to DMSO as a control, GATA1/GAPDH ratio, DMSO versus ACY: 1.5 (±0.35) versus 0.9 (±0.37), *n* = 3, *p* < 0.05. (D) Heat map of Differentially Expressed Genes (DEG) in cells exposed to ACY‐1215 or DMSO (control), *n* = 3. (E) Volcano plot of the 26,897 identified genes (dots). Genes were considered DEG (red dots) with a Log2 fold change threshold of 1 and an adjusted *p*‐value threshold of 0.05 with 1170 upregulated and 786 downregulated. (F) Gene Set Enrichment Analysis (GSEA) of DEG showing enrichment for Bone Marrow Erythroblast (Normalized Enrichment Score (NES) = −7.322, False Discovery Rate (FDR) < 0.001, *p* < 0.001), Heme Metabolism (NES = −6.173, FDR <0.001, *p* < 0.001), GATA1 Targets (NES = −3.238, FDR <0.001, *p* < 0.001), E2F Targets (NES = −3.819, FDR <0.001, *p* < 0.001), Cell Cycle (NES = −2.266, FDR <0.01, *p* < 0.001).

By performing RQ‐PCR analysis to assess key erythroid gene expression, we observed after ACY‐1215 exposure an “immature” signature with an increased expression of *GATA2*, involved in early proliferative phase and a decreased expression of genes associated with late erythroid differentiation such *HBA1* coding for the α‐globin, *EPOR* and *GATA1* (Figure 3A). The decrease in GPA expression observed at cell surface by FCM after ACY‐1215 exposure was confirmed at RNA level. *GATA2/GATA1* ratio, a marker of balance between early proliferation/late differentiation phases, was increased after HDAC6 inhibition (Figure 3B). *GATA1* expression was also slightly reduced at protein level (Figure 3C), showing that ACY‐1215 effect on erythroid differentiation predominates in early ProE before the GATA2 physiological extinction during late erythropoiesis and therefore before completion of the GATA2/GATA1 switch. In order to better understand the transcriptomic modifications induced by ACY‐1215, we performed an RNAseq analysis on D10 erythroblasts exposed to 24 h HDAC6i or DMSO. We observed 1920 DEG including 1134 upregulated and 786 downregulated (Figure 3D,E). Principal component analysis (PCA) clearly separated cells exposed to ACY‐1215 to those exposed to DMSO indicating that HDAC6 inhibition induced a distinct expression pattern (data not shown). Rank‐based GSEA analysis showed a (i) significant enrichment of downregulated genes related to bone marrow erythroblast associated with a downregulation of genes involved in heme metabolism, (ii) consistently with the results obtained by RQ‐PCR, a significant enrichment of downregulated GATA1 targets as well as E2F targets and (iii) an enrichment of downregulated genes related to cell cycle (Figure 3F). Details of RNASeq are presented as a supplemental file.

### 3.4 HDAC6 knockdown in the erythroleukemic cell line UT7/EPO and in erythroid primary cells repressed erythroid differentiation at transcriptional level

**FIGURE 4:**
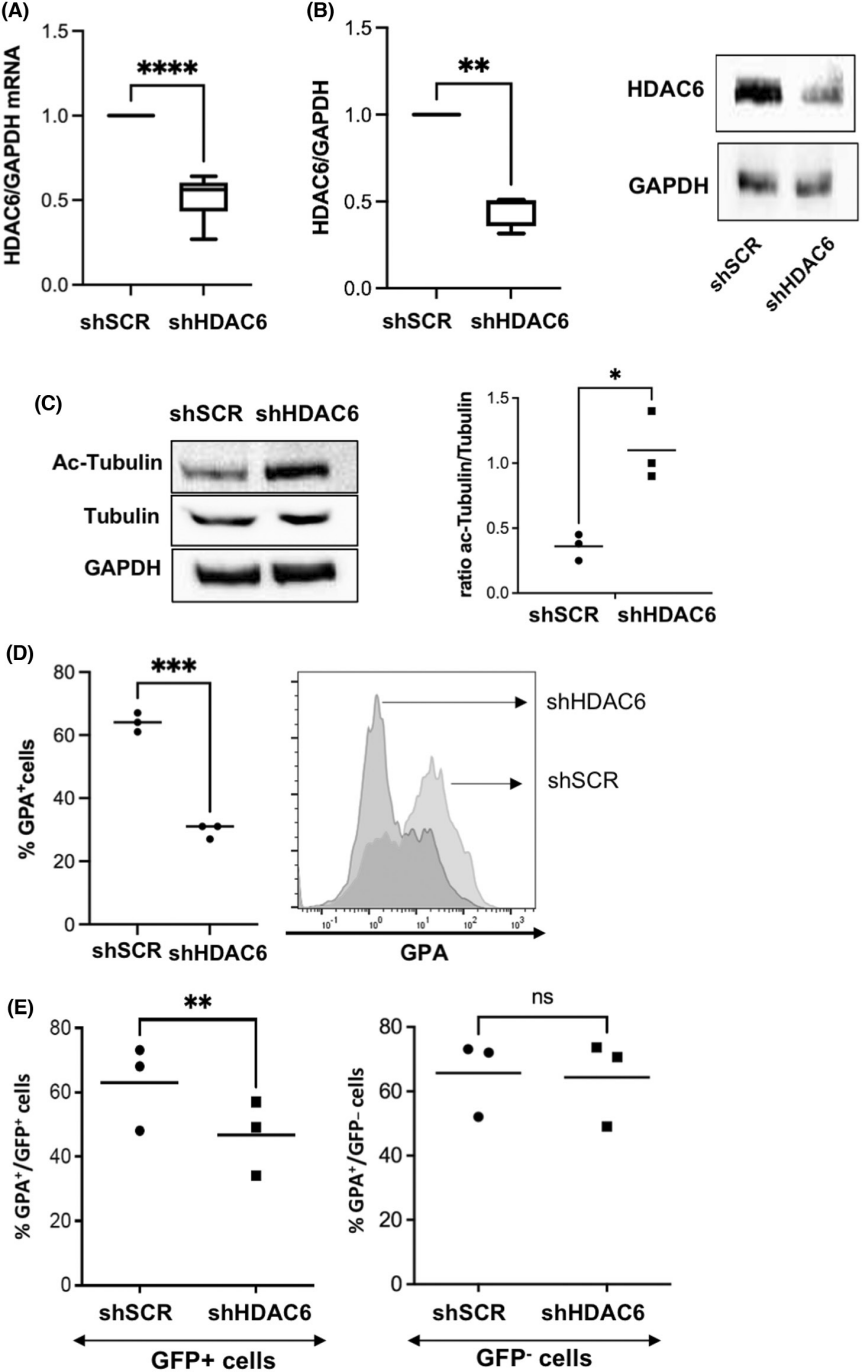
Consequences of shRNA‐mediated HDAC6 knockdown in the UT7/EPO erythroleukemic cell line. (A) HDAC6 knockdown efficiency at RNA level assessed by RQ‐PCR in UT7/EPO cells transduced with a lentivirus encoding a specific shRNA. Mean % of decrease relative to shSCR: 52% (±12), *n* = 8, *p* < 0.0001. (B) HDAC6 knockdown efficiency at protein level assessed by Western blot in UT7/EPO cells. Mean % of decrease relative to shSCR: 45% (±9), *n* = 4, *p* < 0.01. One representative experiment is shown on the right (*n* = 4). (C) α‐tubulin hyperacetylation in UT7/EPO cells transduced with the shHDAC6 lentiviral vector. One representative experiment is shown (*n* = 3). GAPDH was used as housekeeping protein. Ratio acetyl tubulin/tubulin shSCR versus shHDAC6: 0.3 (±0.1) versus 1.1 (±0.26), *n* = 3, *p* < 0.01. (D) Decreased GPA expression at cell surface in UT7/EPO cells expressing a HDAC6 shRNA: percentage of GPA^+^ cells shSCR vs. shHDAC6: 64% (±3) versus 29.7% (±2.3), *n* = 3, *p* < 0.001. Histogram from one representative experiment is shown on the right. (E) HDAC6 knockdown in primary CD34^+^ cells driven into erythroid differentiation led to a decreased GPA expression restricted to GFP+ cells, confirming the data observed in UT7/EPO with the same knock‐down and data obtained using ACY‐1215 in primary cells. Right: one representative experiment, %GPA^+^ cells in the GFP^+^ fraction, shSCR versus shHDAC6: 64% (±13.2) versus 46.7% (±11.7), *n* = 3, *p* < 0.0.1. %GPA^+^ cells in the GFP^−^ fraction, shSCR versus shHDAC6: 66% (±12) versus 64% (±13), *n* = 3, *p* = NS.

In order to rule out any off‐target effect of HDAC6i, we used a shRNA‐mediated HDAC6 knockdown in the erythroleukemic cell line UT7/EPO, a cell line which proliferates in the presence of EPO and expresses a high basal level of HDAC6.^21^, ^24^ This shRNA strategy induced a 50% decrease in HDAC6 expression at RNA and protein level (Figure 4A,B). HDAC6 knockdown led to α‐tubulin hyper acetylation similarly to what was observed after ACY‐1215‐mediated chemical inhibition in primary cells (Figure 4C). HDAC6 shRNA‐mediated knockdown did not induce apoptosis in UT7/EPO cells as shown by Annexin‐V staining (Figure S3A) but decreased GPA expression at cell surface, as assessed by FCM (Figure 4E) and at RNA level (Figure S3B), consistently with what was observed in primary cells using ACY‐1215. HDAC6 knockdown in D10 erythroid primary cells obtained after a double transduction by shHDAC6 expressing lentiviruses led also to a decreased GPA expression that was restricted to the transduced GFP^+^ cells and not significant in the GFP^−^ fraction and to cells transduced with the shSCR as a control (Figure 4E).

### 3.5 HDAC6 knockdown in UT7/EPO and its inhibition in erythroid primary cells inhibited JAK2 phosphorylation in response to EPO stimulation

**FIGURE 5:**
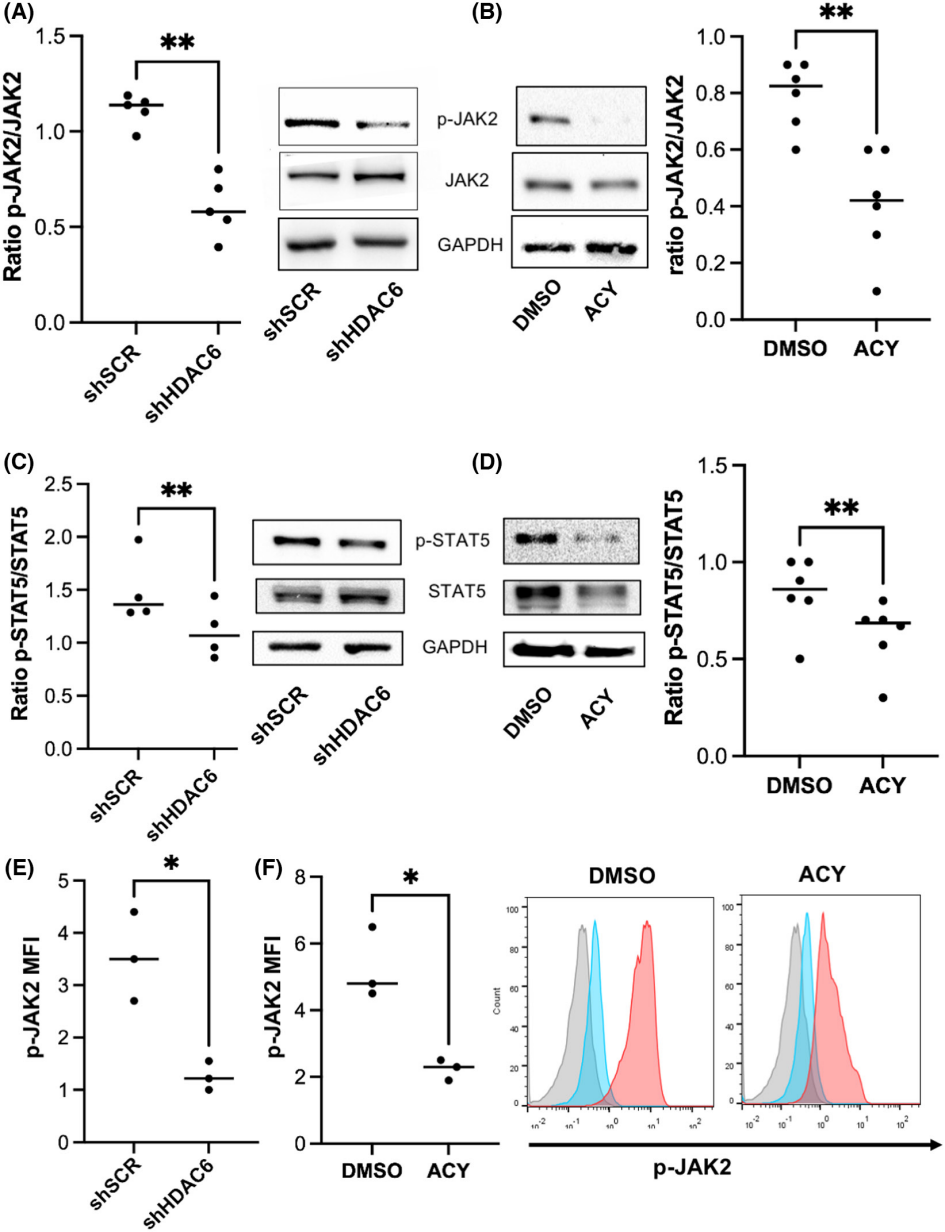
HDAC6 knockdown and chemical inhibition in UT7/EPO cells and primary cells leads to decreased JAK2 and STAT5 phosphorylation in response to EPO. (A) Left: p‐JAK2/JAK2 ratio in UT7/EPO cells assessed by immunoblot after EPO stimulation between shSCR and shHDAC6‐transduced cells: Mean ratio p‐JAK2/JAK2: shSCR versus shHDAC6: 1.11 (±0.08) versus 0.60 (±0.16), *n* = 5, *p* < 0.01. GAPDH was used as housekeeping protein. Right: one representative experiment is shown. (B) Right: p‐JAK2/JAK2 ratio in UT7/EPO cells assessed by immunoblot after EPO stimulation in UT7/EPO cells exposed to DMSO or 0.5 μM ACY‐1215: Mean ratio p‐JAK2/JAK2: DMSO vs. ACY: 0.79 (±0.12) versus 0.41 (±0.19), *n* = 6, *p* < 0.01. GAPDH was used as housekeeping protein. Left: one representative experiment is shown. (C) Left: p‐STAT5/STAT5 ratio in UT7/EPO cells assessed by immunoblot after EPO stimulation in shSCR and shHDAC6‐transduced cells; Mean ratio p‐STAT5/STAT5 shSCR versus shHDAC6: 1.5 (±0.32) versus 1.11 (±0.26), *n* = 4, *p* < 0.01. GAPDH was used as housekeeping protein. Right: one representative experiment is shown. (D): Right: p‐STAT5/STAT5 ratio assessed by immunoblot after EPO stimulation in UT7/EPO cells exposed to DMSO or 0.5 μM ACY‐1215: Mean ratio p‐STAT5/STAT5 DMSO versus ACY: 0.84 (±0.19) versus 0.62 (±0.17), *n* = 6, *p* < 0.01. GAPDH was used as housekeeping protein. Left: one representative experiment is shown. (E) Left: p‐JAK2/JAK2 ratio after EPO stimulation in shSCR and shHDAC6‐transduced UT7/EPO cells assessed by phosphoflow: Mean p‐JAK2 MFI shSCR vs. shHDAC6: 3.5 ± 0.9 versus 1.3 ± 0.3, *n* = 3, *p* < 0.01. (F) Left: p‐JAK2/JAK2 ratio after EPO stimulation in human primary erythroblasts cells exposed to DMSO or 0.5 μM ACY‐1215 assessed by phosphoflow: Mean p‐JAK2 MFI DMSO vs. ACY: 5.2 ± 1.1 versus 2.2 ± 0.3, *n* = 3, *p* < 0.01, *n* = 3. Right: histograms from one representative experiment: grey: isotype, blue: after EPO starvation, pink: after EPO restimulation.

We used the UT7/EPO cell model to study the JAK2/STAT5 response to EPO stimulation after HDAC6 knockdown or chemical inhibition. In all experiments, cells were starved for EPO overnight then restimulated with EPO; JAK2 and STAT5 phosphorylation was assessed using phosphoblot or phosphoflow at 5 minutes. HDAC6 inhibition decreased JAK2 phosphorylation in response to EPO (Figure 5A,B) as well as STAT5 activation (Figure 5C,D), as assessed using immunoblot. We then assessed the JAK2 response to EPO in human primary cells, by flow cytometry. We first validated this method in UT7/EPO cells and confirmed the decreased JAK2 activation in response to EPO in the same range than observed using immunoblot (Figure 5E). In primary cells, we observed a decreased p‐JAK2 mean fluorescence intensity (MFI) after EPO stimulation, when cells were exposed to ACY‐1215 in comparison with DMSO, confirming a defective JAK2 signalling downstream EPO‐R stimulation in both cell models (Figure 5F). Finally, in order to evaluate whether the decreased erythroid differentiation still occurred in cells expressing JAK2^V617F^, we treated HEL cells with DMSO or increasing doses of ACY‐1215. We observed a dose‐dependent decrease in GPA expression, which however occurred at higher doses (4 μM) than in primary cells, while cell death was statistically significant only from 6 μM (Figure S4 **)**.

### 3.6 14.3.3ζ acetylation regulation through HDAC6 activity regulates the JAK2/LNK interactions in human erythroid cells

**FIGURE 6:**
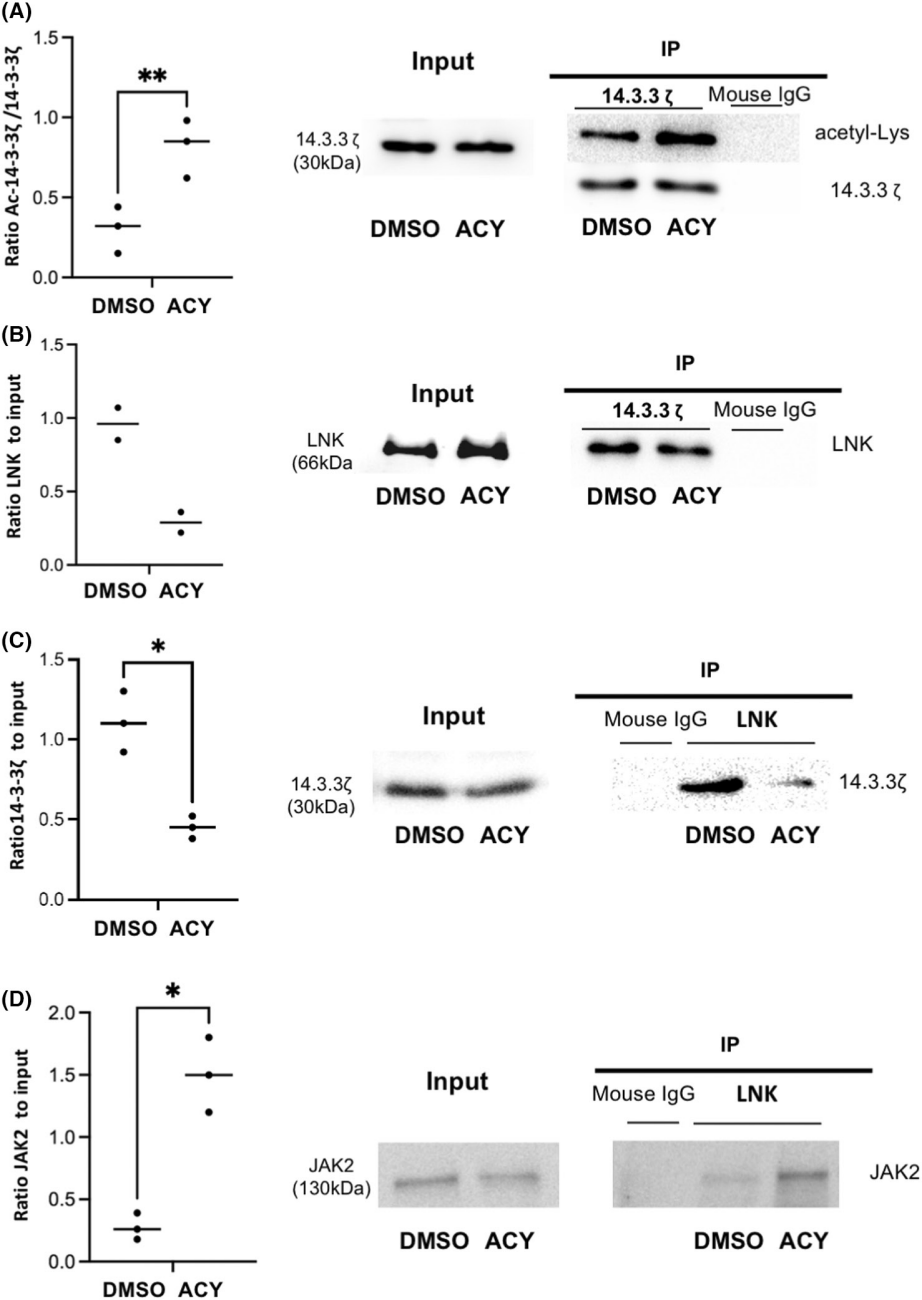
HDAC6 inhibition in human primary erythroblasts leads to 14‐3‐3ζ hyperacetylation, decreased LNK/14‐3‐3ζ and increased JAK2/LNK interaction. (A) 14‐3‐3ζ acetylation level assessed after 14‐3‐3 immunoprecipitation and revelation using an anti‐acetyl lysine antibody in human primary erythroblasts after DMSO or 0.5 μM ACY‐1215 exposure. Left: ratio acetyl/total 14‐3‐3ζ: DMSO versus ACY: 0.30 (±0.146) versus 0.82(±0.18), *n* = 3, *p* = 0.002. Right: one representative experiment is shown. (B) 14‐3‐3ζ/LNK interaction assessed using 14‐3‐3ζ immunoprecipitation and revelation using an anti‐LNK antibody in human primary erythroblasts after DMSO or 0.5 μM ACY‐1215 exposure. Left: summary of two independent experiments, DMSO versus ACY: 0.96 versus 0.29. Right: One representative experiment is shown. (C) 14‐3‐3ζ/LNK interaction assessed using LNK immunoprecipitation and revelation using an anti‐14‐3‐3ζ antibody in human primary erythroblasts after DMSO or 0.5 μM ACY‐1215 exposure. Left: ratio 14‐3‐3ζ after IP/total 14‐3‐3ζ: DMSO versus ACY: 1.11 (±0.19) versus 0.45(±0.07), *n* = 3, *p* < 0.05. Right: one representative experiment is shown. (D) JAK2/LNK interaction assessed using LNK immunoprecipitation and revelation using an anti‐JAK2 antibody in human primary erythroblasts after DMSO or 0.5 μM ACY‐1215 exposure. Left: ratio JAK2 after IP/total JAK2: DMSO versus ACY: 0.28 (±0.1) versus 1.5 (±0.3), *n* = 3, *p* < 0.05. Right: one representative experiment is shown.

In order to identify HDAC6 targets in human erythropoiesis, we developed a “candidate” approach selecting potential targets according to published data and to our own findings. We selected initially two candidates: Survivin and HSP90. Survivin has been involved during murine erythropoiesis and has been reported to be an HDCA6 target.^30^, ^31^ HSP90 is also a known HDAC6 target. Its role in erythropoiesis has been recently reported, through stabilization of immature, heme‐free Hb‐β and Hb‐γ.^32^ However, none of these two candidates were hyperacetylated in UT7/EPO cells after HDAC6 knockdown, ruling out their involvement in the erythroid delay observed in human cells (data not shown). We then focused on 14‐3‐3ζ since (i) 14‐3‐3ζ is a known HDAC6 targets in HEK cells and MDA‐MB‐231 breast cell line^33^ (ii) 14‐3‐3ζ interacts with LNK, a negative regulator of JAK2‐mediated EPO signalling.^34^ We assessed first its acetylation level in primary cells after ACY‐1215 exposure. We performed 14‐3‐3ζ immunoprecipitation followed by Western Blot using (i) an anti‐acetyl lysine antibody and (ii) and anti‐14‐3‐3ζ antibody as a load control. HDAC6 knockdown induced 14‐3‐3ζ hyperacetylation (Figure 6A). We analysed LNK/14‐3‐3ζ direct interactions first using 14‐3‐3ζ immunoprecipitation and revelation with an anti‐LNK antibody. As shown in Figure 6B, ACY‐1215 decreased the 14‐3‐3ζ /LNK interaction. We then immunoprecipitated LNK and compared its interactions with JAK2 and 14‐3‐3ζ before and after ACY‐1215 exposure. We observed that ACY‐1215 decreased LNK/14‐3‐3ζ interaction (Figure 6C) and adversely increased the LNK/JAK2 one (Figure 6D). Taken together, these data showed that HDAC6 inhibition released LNK from its interaction with 14‐3‐3ζ, increasing its availability to interact with and inhibit JAK2.

## 4 DISCUSSION

Since expression and activity of HDACs are altered in many types of cancers,^35^ these enzymes appear to be interesting therapeutic targets, leading to the development of numerous chemical inhibitors. However, the majority of HDACi are non‐selective, inhibiting multiple HDAC isozymes and have toxic off‐target effects due to this broad activity. Among them, haematological toxicity including anaemia is one of the most frequent.^12^, ^13^, ^14^, ^15^, ^19^, ^20^ Several reports highlighted the role of HDACs in mice and human erythropoiesis. The HDACi FK228 and TSA decreased EPO‐erythroid differentiation and induced apoptosis in a model of in vitro differentiation of human CD34^+^ cells, although neither the type of targeted HDAC nor the underlying mechanism was identified.^36^ HDAC1, a class I HDAC, regulates erythroid transcriptional program by interacting among corepressor complexes with transcription factors such as GATA1, Gfi‐1B, TAL‐1, PU‐1, BCL‐11a and KLF1, regulating their availability and activity during the erythroid differentiation.^37^, ^38^, ^39^, ^40^, ^41^, ^42^ The HDAC1/2 KO in haematopoietic cells is lethal in mice, leading to severe haematopoietic defects particularly predominant in the erythroid lineage with decreased maturation and increased apoptosis.^43^, ^44^ HDAC2, another class I HDAC, plays a role in nuclear condensation and enucleation of late erythroblasts.^45^ Among class II HDAC, HDAC5 directly interacts with GATA1, repressing its transcriptional activity and differentiation of MEL cells.^46^ EPO alleviates GATA1 from this complex, leading to its acetylation, while HDAC5^−/−^ progenitors present enhanced erythroid differentiation.^47^ HDAC5 is part of the NuRSERY complex containing pERK and the transcription factors GATA1 and KLF1, favouring their nuclear import.^48^ HDAC5 deficiency impairs human erythroid differentiation through p53 and histone H4 hyperacetylation.^49^

**FIGURE 7:**
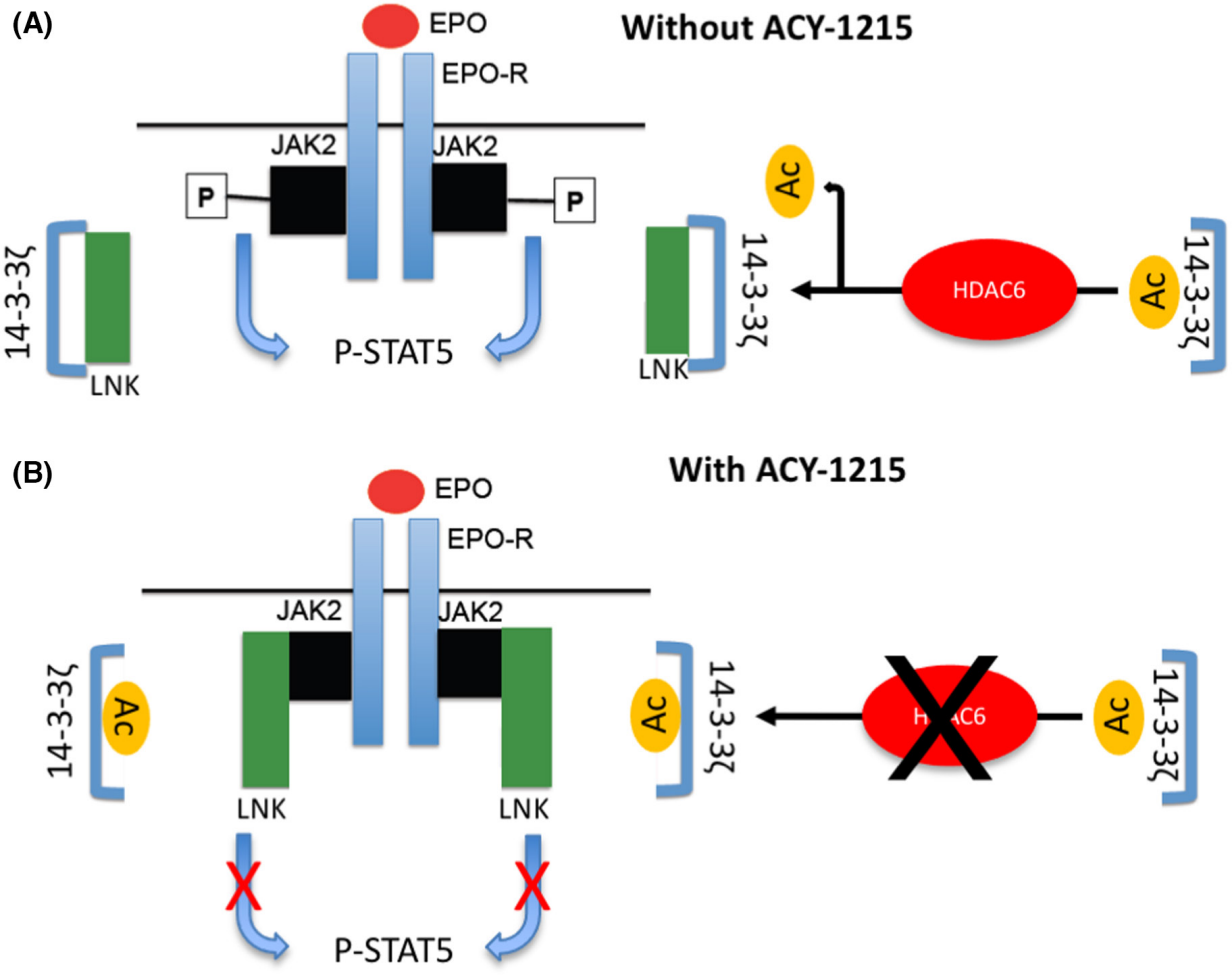
Scheme representing the effect of ACY‐1215 on JAK2 signalling downstream EPO‐R activation. (A) Without ACY‐1215, 14‐3‐3ζ/LNK interactions release JAK2 from the negative control mediated by LNK on its activation and signalization. (B) In the presence of ACY‐1215, hyperacetylation of 14‐3‐3ζ blocks 14‐3‐3ζ/LNK interaction, increasing the LNK fraction that can interact with and inhibit JAK2.

In the treatment of haematological malignancies, a key target among HDACs is class IIb HDAC6. Indeed, inhibition of HDAC6 induces the hyperacetylation of one of its well‐identified targets, the chaperon protein HSP90, which plays an important role in the maintenance and functionality of key regulators of malignant haematopoiesis including mutant FLT3, Bcr‐Abl fusion protein, and in the regulation of signalling proteins such as AKT, c‐Raf and JAK2.^50^ The ability of HDACi to regulate JAK2‐dependant signalling pathways has been largely explored in myeloproliferative neoplasms carrying the constitutively active V617F mutation. Indeed, pan‐HDAC inhibitors downregulate JAK2 V617F protein levels as well as the signalling pathways downstream, both in leukaemic cell lines and in primary cells from patients.^51^, ^52^ HSP90 hyperacetylation decreased its chaperon properties, inducing degradation of its target such as JAK2V617F.^52^ Considering the crucial role of JAK2 during erythropoiesis, it is therefore tempting to hypothesize that the anaemia observed in patients treated with HDAC6i in vivo, and the defect in erythroid differentiation that we observed in vitro are related to an altered HSP90/JAK2 axis. However, in the UT7 leukaemic cell line and in human primary erythroid cells, both expressing a WT JAK2, we noticed that the total level of JAK2 remained unchanged, whereas its phosphorylation in response to EPO was decreased. Besides, HSP90 acetylation level was not modified after HDAC6 inhibition in UT7/EPO cells. This may be related to (i) a different response of JAK2WT and JAK2 V617F to HDACi^52^ (ii) a different mechanism of JAK2 inhibition depending on the HDACi used. Indeed, although HDAC6‐mediated HSP90 acetylation level was thought to mediate JAK2 stability and activity, a recent report identified HDAC11 as the main target to control the JAK–STAT pathway, cell survival and proliferation in leukaemic cells carrying the JAK2V617F mutation and in MPN induced in mice by the MPL^W515L^ mutation.^53^ Since the decreased JAK2 response to EPO and the erythroid differentiation delay observed after HDAC6 inhibition were independent of HSP90 hyperacetylation and JAK2 degradation, it is likely that HDAC6 regulates JAK2 signalling and erythroid differentiation through alternative pathways. We first assessed its cellular localization in erythroid cells. Indeed HDAC6 carries a nuclear localization sequence (NLS) allowing its translocation to the nucleus where it could impact histone acetylation level and control gene expression.^54^ In UT7/EPO and primary erythroid cells, HDAC6 mainly localized in the cytoplasmic compartment and its inhibition did not significantly modify histone H3 acetylation level. Therefore, HDAC6 role in human erythroid differentiation seems mostly related to post‐translational modifications by deacetylation of cytoplasmic substrates. We identified 14‐3‐3ζ as potentially hyperacetylated in primary human erythroid cells after ACY‐1215 exposure, and this was confirmed using immunoblot. HDAC6 ability to deacetylate lysines in the binding pocket of 14‐3‐3ζ has already been described in HEK and MDA‐MB‐231 breast cell lines.^33^ 14‐3‐3 proteins are a family of ubiquitous proteins lacking any intrinsic enzymatic property but able to interact with partners phosphorylated on serine–threonine residues and thereby to modulate their activity through distinct mechanisms such as modification of their conformational state, catalytic activity, cellular distribution or stability.^55^, ^56^ They have been involved through interactions with signalling proteins in the control of cell proliferation, metabolism and survival. 14‐3‐3 proteins are frequently overexpressed in different types of cancer and their knockdown sensitizes malignant cell lines to apoptosis and inhibits cell growth.^57^, ^58^ 14‐3‐3 is regulated by post‐translational modifications, including acetylation. In prostatic cancer cell line, 14‐3‐3ζ hyperacetylation after HDAC6i leads to ERK and CDC25 activation, blockage in cell cycle and apoptosis.^59^ 14‐3‐3ζ is a crucial actor in haematopoiesis through different mechanisms, dependent on cell lineage. In progenitors, it regulates signal transduction downstream of cytokine receptors such as GM‐CSF‐R or IL3‐R.^60^ During murine stress erythropoiesis, 14‐3‐3ζ inhibits FoxO3 nuclear translocation leading to a decreased expression of anti‐oxidant genes.^61^ Moreover, upregulation of 14‐3‐3ζ in mice following knock‐out of its regulator miR‐451 impaired erythropoiesis through increased sensitivity to oxidative stress, and the induced defect in erythropoiesis has been rescued upon 14‐3‐3ζ knock‐down.^62^ We hypothesized that 14‐3‐3ζ was the main HDAC6i target in human erythroid cells. Jiang et al. reported, in murine haematopoietic stem cells, a direct interaction between LNK and 14‐3‐3ζ alleviating the negative control of LNK on JAK2 signalling, although the underlying mechanism was not described.^63^ The negative regulation of JAK2 activity by LNK is crucial, as shown by faster development of MPN in *LNK* ^*−/−*^ mice expressing a mutated JAK2, and by the description of cases of MPN associated with LNK mutations in humans.^64^, ^65^ Here, we show that LNK availability to interact with JAK2 in erythroid cells is regulated by HDAC6‐dependent 14‐3‐3ζ acetylation level. Indeed, ACY‐1215 exposure led to a higher 14‐3‐3ζ acetylation level, lowering its interaction with LNK. In contrast, after HDAC6 inhibition, LNK interacts more strongly with JAK2, leading to the repression of JAK2 signalling in response to EPO and inhibition of erythroid differentiation **(**Figure 7 **)**. Moreover, repression of GPA through HDAC6 inhibition was also observed in HEL cells, arguing indirectly for the relevance of this mechanism in a context of JAK2^V617F^mutation. Thus, in addition to its previously described role in the enucleation mice fetal liver erythroblasts by deacetylation of mDia2,^22^ we identified here a new mechanism of HDAC6‐dependent control of human erythropoiesis through 14‐3‐3ζ acetylation level, LNK availability and finally JAK2 activation in response to EPO.

## AUTHOR CONTRIBUTIONS

**Pascal Vong:** Formal analysis (equal); investigation (lead); writing – original draft (supporting). **Kahia Messaoudi:** Conceptualization (supporting); formal analysis (equal); investigation (lead); methodology (equal). **Nicolas Jankovsky:** Formal analysis (supporting); investigation (supporting); software (supporting); visualization (supporting). **Cathy Gomila:** Investigation (supporting). **Yohann Demont:** Formal analysis (supporting). **Alexis Caulier:** Conceptualization (supporting); writing – review and editing (supporting). **Guillaume Jedraszak:** Formal analysis (supporting); investigation (supporting). **Julien Demagny:** Investigation (supporting). **Stefan Djordjevic:** Investigation (supporting). **Thomas Boyer:** Conceptualization (supporting); formal analysis (supporting); writing – review and editing (supporting). **Jean Pierre Marolleau:** Conceptualization (supporting); methodology (supporting); writing – review and editing (supporting). **Jacques Rochette:** Conceptualization (supporting); formal analysis (supporting); methodology (supporting); validation (supporting); writing – review and editing (supporting). **Hakim Ouled‐Haddou:** Conceptualization (equal); formal analysis (equal); investigation (lead); methodology (equal); writing – original draft (lead); writing – review and editing (equal). **Loïc Garcon:** Conceptualization (lead); funding acquisition (lead); methodology (lead); validation (equal); writing – original draft (lead); writing – review and editing (equal).

## CONFLICT OF INTEREST

Authors have no conflict of interest to disclose.

## Supporting information


Figure S1

Figure S2

Figure S3

Figure S4

Table S1–S3

## Data Availability

Data available on request from the authors.
