# Supplementary figures and images for: HDAC6 regulates human erythroid differentiation through modulation of JAK2 signalling
Source: J Cell Mol Med. 2022 Dec 28;27(2):174–88. doi: 10.1111/jcmm.17559 (PMC9843532; doi:10.1111/jcmm.17559)

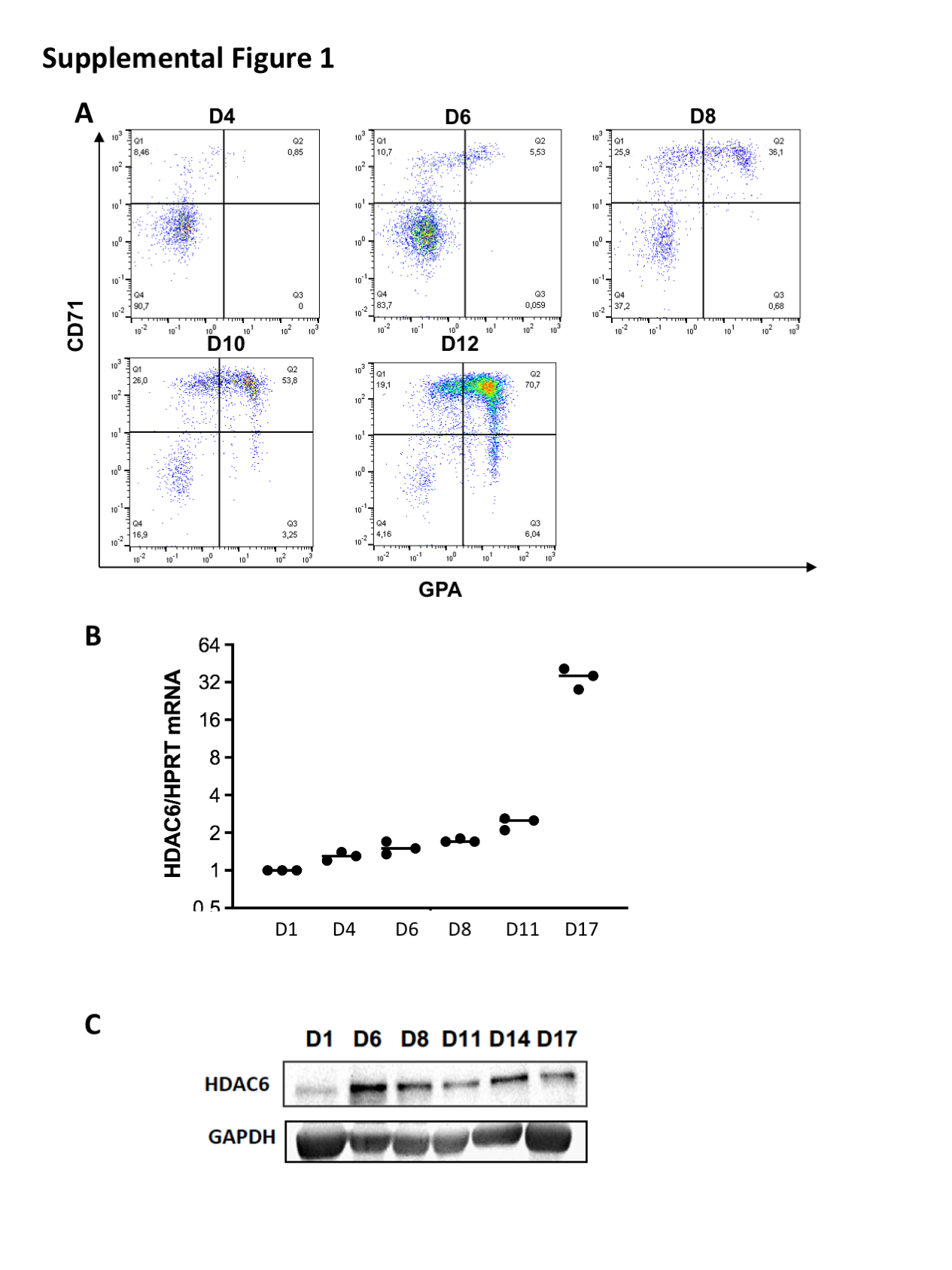

Supplement: Supplementary file 1 — Figure S1 [file JCMM-27-174-s003.tiff]

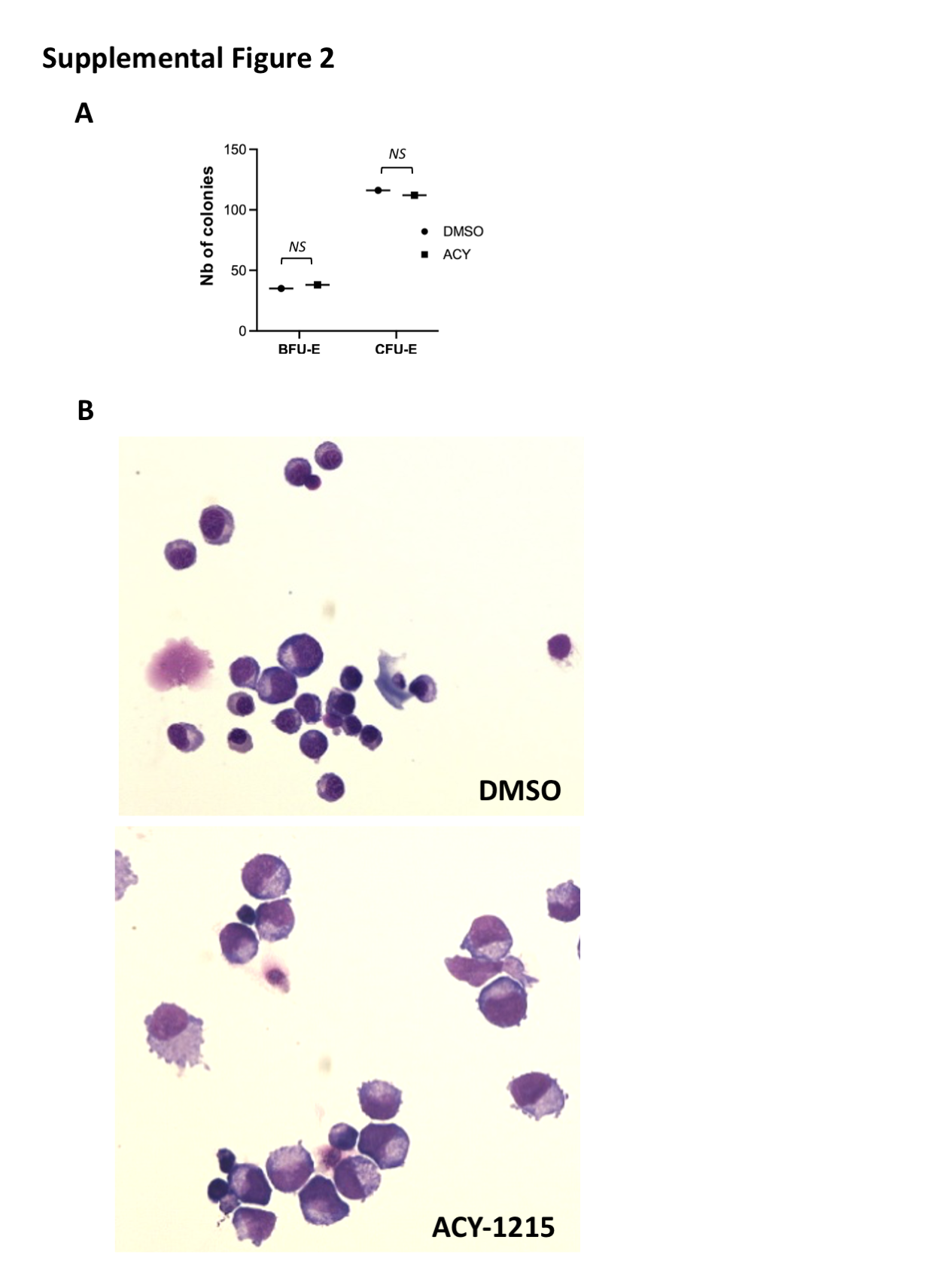

Supplement: Supplementary file 2 — Figure S2 [file JCMM-27-174-s005.tiff]

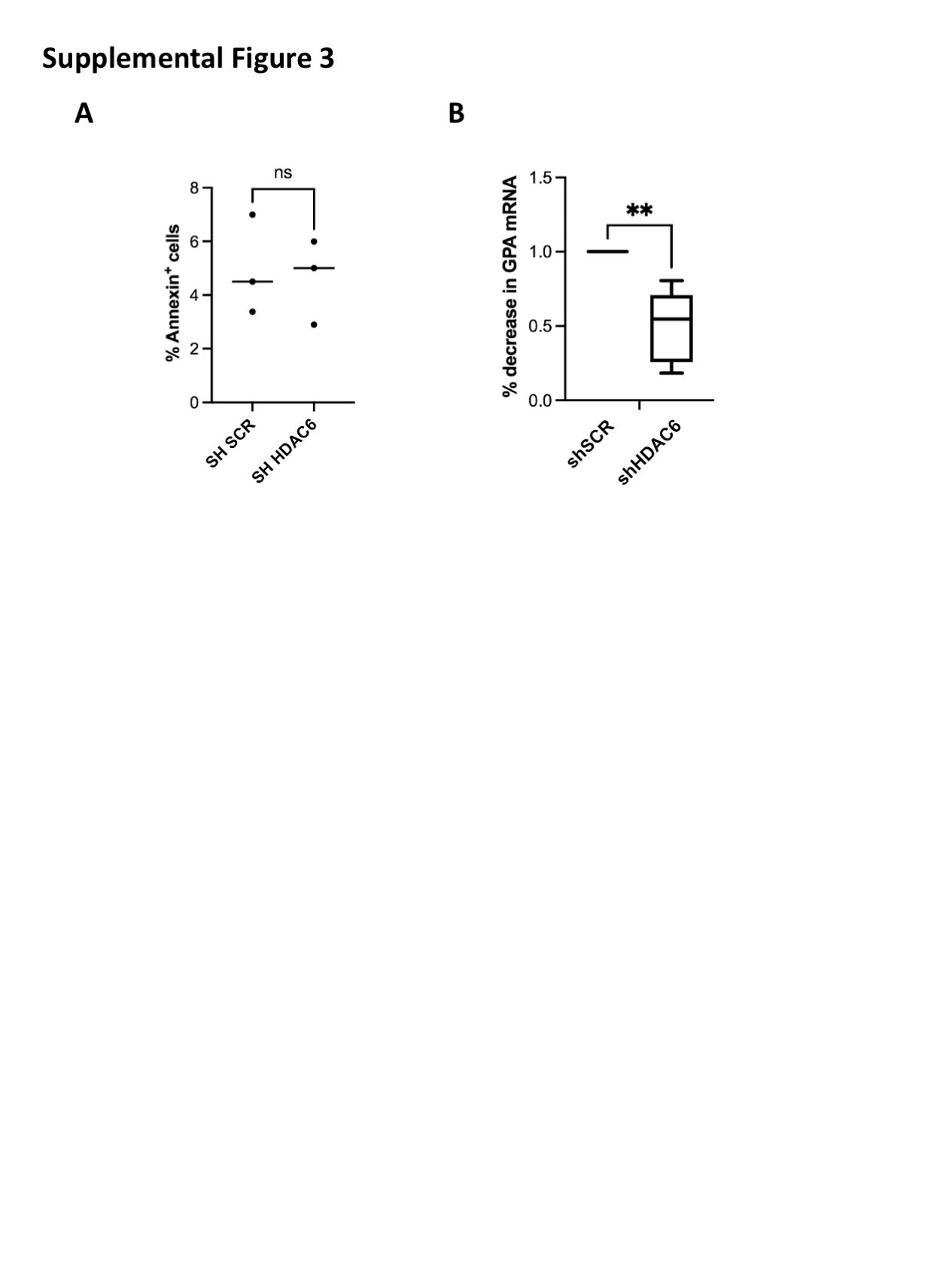

Supplement: Supplementary file 3 — Figure S3 [file JCMM-27-174-s001.tiff]

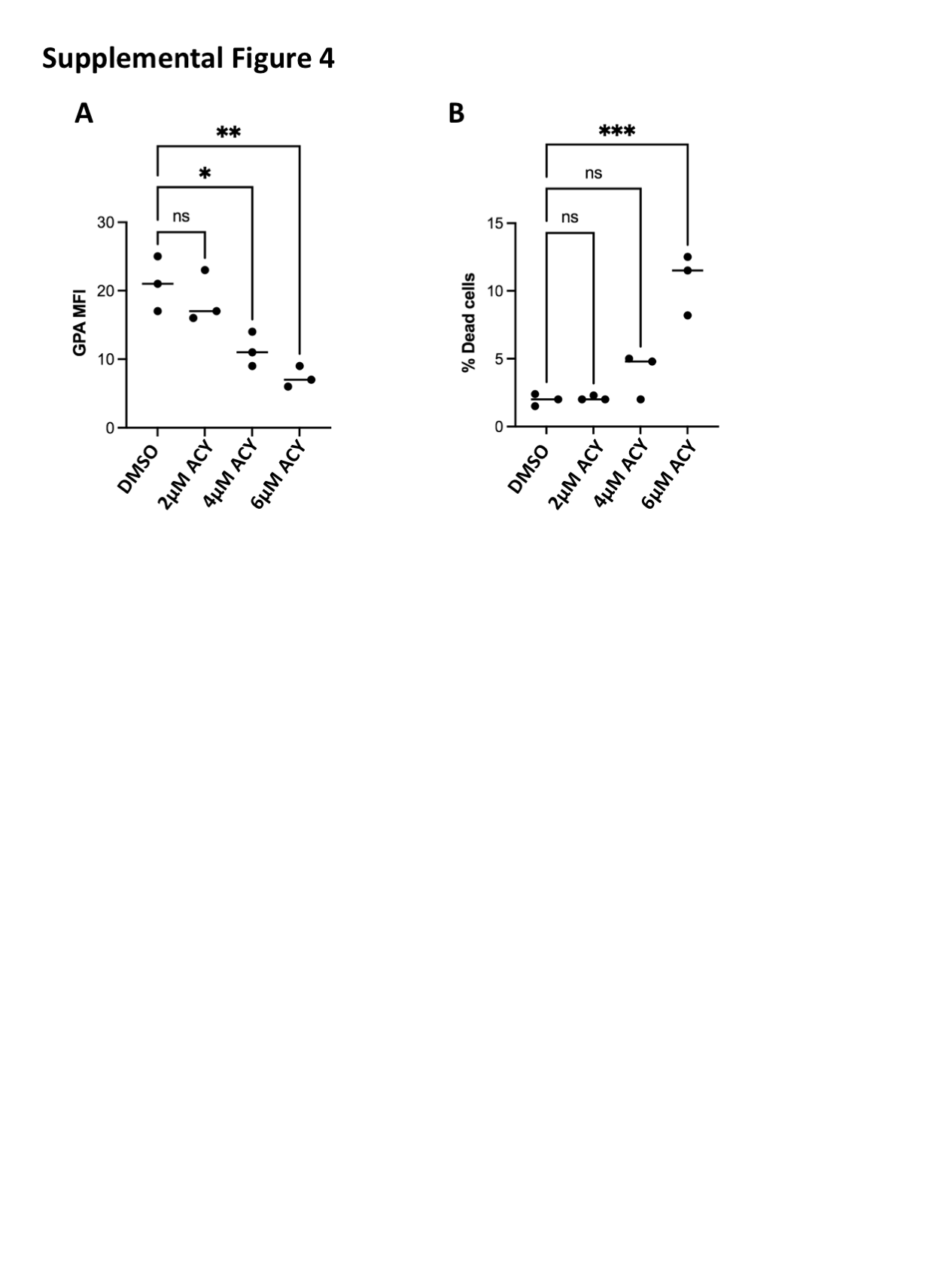

Supplement: Supplementary file 4 — Figure S4 [file JCMM-27-174-s004.tiff]
