# Supplementary material for: HDAC6 regulates human erythroid differentiation through modulation of JAK2 signalling
Source: J Cell Mol Med. 2022 Dec 28;27(2):174–88. doi: 10.1111/jcmm.17559 (PMC9843532; doi:10.1111/jcmm.17559)
Supplement: Supplementary file 5 — Table S1–S3 [file JCMM-27-174-s002.doc]

**Supplemental Tables**

**Supplemental table 1: list of antibodies and dyes used for Flow Cytometry**

| **Antibodies targets** | **Antibodies species** | **References (clones)** |
| --- | --- | --- |
| Anti-hCD235 (GPA) APC | Recombinant human monoclonal | Miltenyi 130-118-356 (REA175) |
| Anti-hCD71 PE | Recombinant human monoclonal | Miltenyi [130-115-029](https://www.miltenyibiotec.com/FR-en/products/cd71-antibody-anti-human-reafinity-rea902.html#copy-to-clipboard) (REA902) |
| Anti-human P-Jak2 (Y1007/Y1008) AF-647 | Rabbit monoclonal | Abcam ab200340 (E132) |

| **Dyes** | **References** |
| --- | --- |
| Annexin V - APC | BD 550475 |
| DAPI | Miltenyi 130-111-570 |
| 7-AAD | BD 559925 |

**Supplemental table 2: list of antibodies used for Western Blot and coIP**

| **Antibodies targets** | **Antibodies species** | **References** |
| --- | --- | --- |
| Anti-human GAPDH | mouse | Santacruz (32233) |
| Anti-human Actine | mouse | Sigma (A1978) |
| Anti-human Acetylated α-tubuline | Mouse | Santacruz (23950) |
| Anti-human α-tubulin | Mouse | Santacruz (5286) |
| Anti-human Acetylated histone H3 | Mouse | Santacruz (56616) |
| Anti-human histone H3 | mouse | Sancruz (517576) |
| Anti-human HDAC6 | mouse | Santacruz (28386) |
| Anti-human GATA-1 | rabbit | Abcam (173816) |
| Anti-human HSP90 | mouse | Santacruz (13119) |
| Anti-human survivin | mouse | Santacruz (17779) |
| Anti-human P-Jak2 (Y1007/1008) | rabbit | Cellsignaling (3771S) |
| Anti-human Jak2 | rabbit | Cellsignaling (3230S) |
| Anti-human P-Stat5 (Y694) | rabbit | Cellsignaling (9351S) |
| Anti-human P-Stat5 a/b (Ser726-R) | rabbit | Santacruz (12893-R) |
| Anti-human Stat5 a/b | mouse | Bd Biosciences |
| Anti-human pan 14-3-3 | mouse | Santacruz (1657) |
| Anti-human LNK | mouse | Santacruz (393709) |
| Anti-human Acetylated lysine | rabbit | Cellsignaling (9814) |
| igG isotype control | Mouse | Santacruz (2025) |
| igG isotype control | Rabbit | Abcam (37415) |

| **Targeted Species** | **Antibodies species** | **References** |
| --- | --- | --- |
| mouse | Goat-HRP | Santacruz (2005) |
| rabbit | Goat-HRP | Santacruz (2301) |
| rat | Goat-HRP | Abcam (97057) |

**Supplemental table 3: list of primers used for RQ-PCR**

All primer pairs were designed to span an exon–exon boundary using Primer Blast tool, purchased by Eurogentec and validated for their specificity.

| **Genes** | **Primers 5’ -> 3’** |
| --- | --- |
| GAPDH-FOR GAPDH-REV | AAGGTGAAGGTCGGAGTCAA  CTTGACGGTGCCATGGAATT |
| HDAC6-FOR HDAC6-REV | TGGCTATTGCATGTTCAACCA  GTCGAAGGTGAACTGTGTTCCT |
| GPA-FOR  GPA-REV | GGGGTGATGGCTGGTGTTAT CACGTCTGTGTCAGGTGAGG |
| GATA-1-FOR  GATA1-REV | AGCAGCTTCCTCCACTGC TGGGTACACCTGAAAGACTGG |
| GATA-2-FOR  GATA-2-REV | TACAGCAGCGGACTCTTCC  CTCGTTCCTGTTCAGAAGGC |
| EPO-R-FOR EPO-R-REV | ATCCTGACGCTCTCCCTCAT GAGGCCTTCAAACTCGCTCT |
| HBA1-FOR HBA1-REV | GGTCAACTTCAAGCTCCTAAGCC AAGAAGCATGGCCACCGAGG |
| HBB-FOR HBB-REV | CAACGTGCTGGTCTGTGTG  CAGCAAGAAAGCGAGCTTAGTG |
| ALAS2-FOR ALAS2-REV | AGGATGTGTCCGTCTGGTGTA  TGAAACTTACTGGTGCCTGAGA |
| RPS19-FOR RPS19-REV | CGCGAGCTGCTTCCACA  TCCACCATTTTCAGCCCCTC |
| FOG1-FOR FOG1-REV | CCAGGCGGAAACAGAGCAAC  GAGTTAACATCTGCGCTGGGA |
| KLF1-FOR KLF1-REV | TGACTTCCTCAAGTGGTGGC  GGTGAGGAGGAGATCCAGGT |
| TAL1-FOR TAL1-REV | AGCCGGATGCCTTCCCTAT  GGGACCATCAGTAATCTCCATCT |
| MYB-FOR MYB-REV | TCAGGAAACTTCTTCTGCTCACA  AGGTTCCCAGGTACTGCT |
| BMI1-FOR BMI1-REV | CCACCTGATGTGTGTGCTTTG  TTCAGTAGTGGTCTGGTCTTGT |
| IRF6-FOR IRF6-REV | TGGTAGCGACGGGTGATCTT  CGGCTTGTCTTTCCCTTGAC |
| KIT-FOR KIT-REV | CCCACCCTGGTCATTACAGAA  TACTATCGCTGCAGGAAGACTC |
| AHSP-FOR AHSP-REV | CTGACACTTGACTCCTTGCC  AGAGCCATCTTCAGGTCTAACAG |
| SLC2A1-FOR SLC2A1-REV | TTGGCTCCGGTATCGTCAAC  GGCCACGATGCTCAGATAGG |
| STAT5A-FOR STAT5A-REV | CTGGACTTTTCTGAAGGGGCT  AGAATAGCCGGGGGAATGTG |
| EFE-FOR EFE-REV | TGCTCATCTGCATCCAGTCC  TTGTCCAAGAACACGGAGGTC |
| SLC4A1-FOR SLC4A1-REV | GGGCTCAGATCACCGTAGAC  GTAGTCTGTGGCTGTTGCCT |
| FOXO3-FOR FOXO3-REV | ATCTACGAGTGGATGGTGCG  TGCCAGTTCCCTCATTCTGG |
| FOS-FOR FOS-REV | CCAAGCGGAGACAGACCAAC  ATCAGGGATCTTGCAGGCAG |
| FTL-FOR FTL-REV | CCAGCACCGTTTTTGTGGTT  GGTCGAAATAGAAGCCCAGAGA |
| YWHAZ-FOR YWHAZ-REV | ATGGATAAAAATGAGCTGGTTC  TTAATTTTCCCCTCCTTCTC |
| HPRT-FOR  HPRT-REV | GGCAGTATAATCCAAAGATGGTCAA  TCAAATCCAACAAAGTCTGGCTTATAT |
